# Medicaid Coverage and Emergency Department Utilization in Southeastern Pennsylvania

**DOI:** 10.7759/cureus.45464

**Published:** 2023-09-18

**Authors:** Olusegun Bakare, Ikeoluwa A Akintujoye, Paul E Gbemudu, Rheiner N Mbaezue, Abimbola O Akinbolade, Segun Olopade

**Affiliations:** 1 Internal Medicine-Pediatrics, Tulane University School of Medicine, New Orleans, USA; 2 Public Health and Social Justice, Saint Louis University, Saint Louis, USA; 3 Health, Department of Health, Cape Town, ZAF; 4 Medicine, University of Lagos, Lagos, NGA; 5 Medicine and Surgery, Igbinedion University Teaching Hospital, Okada, NGA

**Keywords:** affordable care act, overutilization, healthcare cost, emergency department, medicaid

## Abstract

Overutilization of the emergency department (ED) is a significant problem in the United States (US), characterized mainly by patients with non-emergent conditions seeking care in a setting designed specifically for acute care. This has significantly increased healthcare costs in the US, a country with one of the most expensive healthcare systems in the world. ED overutilization was also found to be high among people with Medicaid coverage, especially since the Affordable Care Act was enacted with an expansion in Medicaid coverage. Using the 2018 South Eastern Pennsylvania (SEPA) Household Health Survey, we identified a significant bivariate relationship between emergency department visits and the following predictor variables: sex, race, education, employment status, 150% poverty level, and Medicaid recipient. Using a multivariable logistic regression model, Medicaid recipients had higher odds of presenting to the ED than non-Medicaid recipients [odds ratio (OR): 2.863, 95% confidence interval (CI): 2.164, 3.788]. Black people (OR: 1.647, 95% CI: 1.411, 1.923) and Native Americans (OR: 2.985, 95% CI: 1.536, 5.800) had higher odds than Whites. Respondents without a high school diploma had higher odds than college graduates (OR: 1.647, 95% CI: 1.96, 2.273). Respondents below the 150% poverty line had higher odds than those at or above the 150% poverty level (OR: 1.651, 95% CI: 1.386, 1.968). Unemployed respondents had higher odds than full-time employed respondents (OR: 1.703, 95% CI: 1.488, 1.953) or part-time (OR: 1.259, 95% CI: 1.036, 1.529). No difference was observed between the sexes. Addressing ED overutilization should take a multi-faceted approach with the ultimate goal of improving access to primary care.

## Introduction

Overutilization of the emergency department (ED) is a significant problem in the United States (US), characterized mainly by patients with non-emergent conditions seeking care in a setting designed specifically for acute care [1] This has contributed to the high cost of care in the US, making it the country with the most expensive healthcare yet with an unimpressive health outcome for US patients based on life expectancy and disease-specific mortality rates [2]. ED overutilization leads to inadequate provision of preventive care and a lack of proper care coordination, which is essential to ensuring positive health outcomes [3]. Using the ED for non-emergent conditions is a direct cause of missed opportunities to promote longitudinal relationships with primary care physicians [4].

Numerous factors, such as a lack of timely access to primary care providers [1] and low health literacy [5, 6], contribute to this issue. Living in a rural area where service availability differs from that in an urbanized area also plays a role in ED overuse. In addition, social and material deprivation is associated with poor access to preventive care, causing ED overuse [7]. Disparities have also been observed across racial lines - minorities were found to frequent the ED at higher rates than Whites [8].

Medicaid patients utilize the ED at higher rates than patients with private insurance [1, 9, 10]. The Affordable Care Act (ACA) sought to increase healthcare access by allowing states to expand Medicaid, despite concerns that it might increase preventable ED visits [11]. In the United States, about 38 to 41 states, including the District of Columbia, have enacted Medicaid expansion, and as a result, the quantity of patients covered by the program has greatly expanded [12, 13]. One study purported to show that after one year, there were no significant differences in overall ED use between states that expanded Medicaid and those that did not [11]. However, several studies tracking ED utilization over a longer period have disputed that narrative [10, 14, 15]. Nonetheless, the underlying reason Medicaid beneficiaries overutilize the ED remains elusive. While it has been suggested that introducing co-payments would deter patients from using the ED for subacute care [16], these hopes have not materialized [17]. 

To adequately address ED overuse and overcrowding, the underlying factors causing improper usage must be determined first. Using the 2018 Southeastern Pennsylvania (SEPA) Household Health Survey (HHS), we investigated factors influencing ED overuse in Southeastern Pennsylvania. Our main objective is to determine if there is an association between Medicaid coverage and ED use in this population.

Additionally, we explored whether other factors, such as race, education level, and poverty, have possible associations with overutilization of ED use.

## Materials and methods

Data source

The Public Health Management Corporation (PHMC), a non-profit organization, conducted the 2018 SEPA HHS. This survey is usually conducted every two to three years and focuses on the timely collection of health indicators in the SEPA region. The HHS covers about 7,500 households in five counties: Bucks, Chester, Delaware, Montgomery, and Philadelphia. The 2018 HHS was conducted using landline and mobile phone interviews from August 2018 through January 2019. It was centered on health status, personal health behaviors, access to care, and utilization of and quality of health services. The PHMC conducted the survey with residents 18 years of age and older across the five counties. All households reachable by phone were eligible for sample selection. One adult was interviewed in each household, with each interview lasting an average of 20 minutes. The survey excluded the homeless population and incarcerated population. Respondents who completed the interviews were given $5. Institutional Review Board (IRB) approval was not needed as data from the survey is publicly available to institutional subscribers.

Sample

The samples for this HHS survey were recruited by one of three methods: random generation of landline and cellphone numbers belonging to the desired region; voter registration records; and re-contacted respondents from previously administered surveys. The total number of completed surveys was 7,501. Among adults, a total of 3,407 interviews were conducted with those between 18 and 59 years old, while 4,094 interviews were conducted with people over 60 years old. Because some segments of the population are underrepresented in the survey, advanced methods are needed to reduce the weight of overrepresented segments of the population and add weight to underrepresented segments. The PHMC dataset includes balancing weights that should be applied to multivariable analysis to account for these potential sampling errors. The Data set was weighted using the adult balancing weight (ADBALWT).

Measures

We measured the outcome variable as “how many visits by respondents to a hospital emergency room during the last 12 months, quantified as zero visits or one or more visits.” We measure the independent variable using the primary source of health coverage employer or union purchase, self-purchased plan, Medicare, Medicaid, or other state programs, and Tricare, formerly the Civilian Health and Medical Program of the Department of Veterans Affairs (CHAMPVA) for VA or military. The independent variable was recorded into either “Medicaid coverage” or “no Medicaid coverage”. People without insurance coverage were included in the group without Medicaid coverage.

We included covariates from the dataset to include the gender of the respondent (“What is the selected adult’s gender?” (Male or female); race/ethnicity (White, Black, Asian, Latino, Biracial, Native American, and others); and age (18-34, 35-49, 50-64, 65+). We also included education measured as the last grade of school completed (less than high school, high school, technical/vocational training, and college graduate); employment (full-time, part-time, and unemployed); and poverty status (below the 150% poverty line and at or above the 150% poverty line).

Statistical analysis

We analyzed the data using the Statistical Package for the Social Sciences (IBM Corp. Released 2020. IBM SPSS Statistics for Windows, Version 27.0. Armonk, NY: IBM Corp). We calculated descriptive statistics summarizing demographic variables, income, employment status, primary insurance coverage, and the proportion of individuals who have visited the ED at least once in the past year. We used Chi-square tests to assess bivariate associations between ED visits and covariates. Based on those findings, we used a multivariable logistic regression model to analyze the independent association of covariates with ED visits. 

## Results

A total of 7,501 respondents were included in the analysis. 4676 (64.4%) were White/Caucasian, 1423 (19.2%) Black, 232 (3.1%) Asian, 566 (7.6%) Latino, and 244 (3.3%) Biracial, 48 (0.6%) Native American, and 121 (1.6%) Other (Table 1).

**Table 1:** Table 1: Demographic characteristics of adults participating in the 2018 South Eastern Pennsylvania (SEPA) Household Health Survey (HHS).

Demographic Characteristics	Total Participants: N (%)
Race	
White (Not-Latino)	4767 (64.4%)
Black (Not-Latino)	1423 (19.2%)
Latino (total)	566 (7.6%)
Asian	232 (3.1%)
Biracial/Multi	244 (3.3%)
Native American	48 (0.6%)
other	121 (1.6%)
Sex	
Male	3501 (46.7%)
Female	4000 (53.3%)
Age	
18-24	2027 (27%)
35-49	1351 (18%)
50-64	2514 (33.5%)
65+	1609 (21.4%)
Does the respondent have Medicaid coverage?	
Not on Medicaid	6025 (91.28%)
Medicaid Recipient	575 (8.71%)
Poverty status	
Below 150% of the poverty line	1578 (21.11%)
At or above 150% of the poverty line	5896 (78.88%)
Employment Status	
Not currently employed	2702 (37.34%)
Employed full time	3700 (51.15%)
Employed part-time	833 (11.51%)
Education level	
Less than High School	352 (4.72%)
High school graduate	1763 (23.64%)
Some colleges/technical schools	1652 (22.15%)
College Graduate	3689 (49.49%)

Among Medicaid recipients (N = 575), 312 (54.3%) had one or more visits to the ED in the past year, compared with 1486 (24.7%) of those who do not have Medicaid as their primary insurance (N = 6025) (Table 2). We identified a significant bivariate relationship between emergency department visits and the following predictor variables: sex, race, education, employment status, poverty status, and Medicaid recipient. 

**Table 2:** Bivariate Analysis of Factors Associated with Emergency Department Visits in a Year, among Adults Participating in the 2018 SEPA HHS. N: total respondents

Variable (N)	Emergency Room Visits		X^2^	P-Value
	O Visit N (%)	≥ 1 visits N (%)		
RACE (7378)			172.515	<0.001
White (Not-Latino)	3661 (77.10%)	1090 (22.90%)		
Black (Not-Latino)	875 (61.80%)	542 (38.20%)		
Latino (total)	392 (69.40%)	173 (30.60%)		
Asian	191 (82.00%)	42 (18.00%)		
Biracial/Multi	163 (67.10%)	80 (32.90%)		
Native American	20 (41.70%)	28 (58.30%)		
Other	86 (71.10%)	35 (28.90%)		
SEX			19.624	<0.001
Male	2627 (75.40%)	859 (24.60%)		
Female	2824 (70.80%)	1165 (29.20%)		
AGE (years)			4.421	0.219
18-34	1496 (74.00%)	525 (26.00%)		
35-49	973 (72.40%)	371 (27.60%)		
50-64	1842 (73.40%)	666 (26.60%)		
65+	1140 (71.10%)	463 (28.90%)		
Does the respondent have Medicaid coverage? (6600)			231.98	<0.001
Not on Medicaid	4539 (75.30%)	1486 (24.70%)		
Medicaid Recipient	263 (45.70%)	312 (54.30%)		
150% of the poverty level (7474)			222.121	<0.001
Below 150% of the poverty line	917 (58.10%)	661 (41.90%)		
At or above 150% of the poverty line	4533 (76.90%)	1363 (23.10%)		
Employment Status (7235)			186.474	<0.001
Not currently employed	1740 (64.40%)	962 (35.60%)		
Employed full time	2945 (79.60%)	755 (20.4%)		
Employed part time	581 (69.7%)	252 (30.30%)		
Education level (7456)			157.654	<0.001
Less than High School	206 (58.5%)	146 (41.50%)		
High school graduate	1161 (65.9%)	602 (34.10%)		
Some colleges/technical schools	1156 (70%)	496 (30%)		
College Graduate	2914 (79%)	775 (21%)		

In the multivariable logistic regression model (Table 3), Medicaid recipients had higher odds of presenting to the ED than non-Medicaid recipients (OR: 2.863, 95% CI: 2.164, 3.788). Black people (OR: 1.647, 95% CI: 1.411, 1.923) and Native Americans (OR: 2.985, 95% CI: 1.536, 5.800) had higher odds than Whites. Respondents without a high school diploma had higher odds than college graduates (OR: 1.647, 95% CI: 1.96, 2.273). Respondents below the 150% poverty line had higher odds than those at or above the 150% poverty level (OR: 1.651, 95% CI: 1.386, 1.968). Unemployed respondents had higher odds than full-time employed respondents (OR: 1.703, 95% CI: 1.488, 1.953) or part-time (OR: 1.259, 95% CI: 1.036, 1.529). No difference was observed between the sexes. 

**Table 3:** Binary Logistic Regression of Factors Associated with One or More Emergency Department Visits in a Year, among Adults Participating in the 2018 SEPA HHS. (*p < 0.05; **p < 0.001), The Data set was weighted and adjusted using the adult balancing weight (ADBALWT).

Logistic regression variables (reference)	Adjusted odds ratio	95% CI
Medicaid Recipient (not on Medicaid)	2.863**	(2.164, 3.788)
Race: Black (White)	1.647**	(1.411, 1.923)
Race: Native American (White)	2.985*	(1.536, 5.800)
Race: Biracial/multi (White)	1.740*	(1.245, 2.433)
No high school diploma (college graduate)	1.647*	(1.96, 2.273)
Unemployed (Employed full time)	1.703**	(1.488, 1.953)
Unemployed (Employed part-time)	1.259*	(1.036, 1.529)
Below 150% poverty line (at or above 150%)	1.651**	(1.386, 1.968)
Female (male)	.902	(.798, 1.021)
Below 150% poverty line and Medicaid recipient (above 150% poverty line and not on Medicaid)	*.635	(.430, .937)

## Discussion

Our study identified the demographics of Southeastern Pennsylvania residents who visited the ED. We found a significant association between Medicaid coverage and ED use, which is in line with prior studies [1, 9, 11, 18]. 

Racial and educational disparities have also been shown to be associated with increased ED visits [5]. Racial disparity is associated with an increase in ED visits. In comparison to White patients, Black, and Hispanic patients reported using the ED more frequently [19]. Apart from the usage disparity, the level of care is generally poor [19]. Routine ED visits last noticeably longer for non-White patients than for White patients, particularly in non-teaching institutions, where there is a considerable racial imbalance [20]. Hispanic Medicare outpatient beneficiaries reported worse care experiences than White Medicare outpatient beneficiaries, but Hispanic Medicare beneficiaries reported improved care experiences in health plans with a higher proportion of Hispanic enrollees [19].

Education disparity has also been shown to be associated with increased ED visits [5]. 48.8 million U.S. adults are projected to be illiterate [21], and the inability of a parent to understand the process of a child's health concerns will affect the outcome of care. Children with an uncomplicated medical condition and low caregiver health literacy use ED more than those with the same conditions and high caregiver health literacy [22]. According to Griffey et al. [23], those with poor health literacy and new Medicare enrollees use EDs more frequently than those with adequate health literacy, which is a concern. Poor health literacy substantially impairs parents’ capacity to utilize health information and make decisions regarding their child's health [24] as a result, health literacy is a significant barrier to providing adequate and necessary medical services to patients, and that could lead to a recurrent ED visit.

Medicaid recipients face more barriers to accessing primary care services due to lengthy appointment dates, difficulty requesting time off work for doctor's appointments, and the fact that primary care providers' offices are closed on weekends for those who can only visit the clinic on weekends due to their jobs [1]. While this study highlights the characteristics of ED users, it has limitations. The PHMC data was collected via in-depth interviews, making it a very reliable and encompassing sample. However, as with any data collected via surveys, there is the possibility of recall bias. Also, by using data from an existing survey, we could not alter or add questions. The survey excluded the homeless population and incarcerated people, who are also users of the ED. Given the sensitive nature of some of the questions, it is possible that subject-related biases are present as well. Additionally, the subjects' medical records were not included in the survey, and it is possible that patients are on Medicaid because of frail health, thus requiring frequent care and being prone to medical emergencies; this should be included in future studies as it is a possible confounder. Nonetheless, given the size of the study and the care taken to obtain a representative sample, it is unlikely that any of the above factors significantly skewed the data. 

Implications

Interventions to reduce overutilization of the ED should focus primarily on Medicaid patients, as they are more likely to utilize the ED than their peers. Increasing access to primary care should be considered, as a study shows that Medicaid-insured patients have limited access to primary care compared to privately insured patients, thus increasing ED utilization [1]. 

Addressing educational disparities to improve ED overuse may also prove effective. Education has been shown to be the most effective method of preventing frequent ED visits, especially among pediatric patients, as parents with little health literacy are more likely to use the ED than those with extensive health knowledge about their child's illness [5]. Educating caregivers on the types of illnesses and the appropriate location for events reduces ED overutilization [25]. 

Next steps

More studies are required to assess the reason Medicaid patients utilize the ED more often than other patients. Given the educational gap, it would be worthwhile to investigate whether patient education about the proper use of the ED would be an effective intervention. 

## Conclusions

The study found that people with poor health literacy, Medicaid recipients, or those newly covered under Medicaid insurance were significantly more likely to use the ED than those on other insurance plans. Medicaid patients may not be aware of the appropriate settings for care, or they may not have access to other healthcare options. This study provides insight into policymakers and healthcare providers. Policymakers can work to improve access to primary care and other healthcare services for Medicaid patients. Healthcare providers can educate patients about the appropriate use of the ED and provide referrals to other healthcare settings when necessary.

The study also found that racial disparities exist in ED use. Black and Hispanic patients were more likely to use the ED than White patients. This suggests that there may be barriers to accessing healthcare for these populations, such as discrimination or a lack of transportation; suggesting that a multifaceted approach is needed to reduce overutilization of the ED, which should include interventions to improve access to primary care, address racial and educational disparities, and educate patients about the appropriate use of the ED.
